# Using the comprehensive complication index to assess the impact of Global Leadership Initiative on Malnutrition (GLIM)-defined malnutrition on postoperative complications after resection for biliary tract cancer

**DOI:** 10.1007/s00595-025-03051-9

**Published:** 2025-05-27

**Authors:** Yuki Okazoe, Hiroaki Yanagimoto, Daisuke Tsugawa, Masayuki Akita, Takuya Mizumoto, Toshihiko Yoshida, Shinichi So, Jun Ishida, Takeshi Urade, Yoshihide Nanno, Kenji Fukushima, Hidetoshi Gon, Shohei Komatsu, Sadaki Asari, Hirochika Toyama, Masahiro Kido, Takumi Fukumoto

**Affiliations:** Department of Surgery, Division of Hepato-Biliary-Pancreatic Surgery, Kobe University Graduate School of Medicine, 7-5-2 Kusunoki-Cho, Chuo-Ku, Kobe City, Hyogo 650-0017 Japan

**Keywords:** Biliary tract cancer, GLIM criteria, Malnutrition, Postoperative complication

## Abstract

**Purpose:**

The Global Leadership Initiative on Malnutrition (GLIM) criteria, proposed in 2018, provide universal diagnostic standards for malnutrition, a known risk factor for postoperative complications in patients with various cancers. However, its impact on surgery for biliary tract cancer (BTC) remains unclear. This study evaluates the relationship between GLIM-defined malnutrition and postoperative complications after resection for BTC.

**Methods:**

The subjects of this retrospective study were patients who underwent pancreaticoduodenectomy or major hepatectomy with extrahepatic bile duct resection for BTC between January, 2013 and December, 2021. The comprehensive complication index (CCI), an indicator of postoperative complications, was calculated based on the total number and severity of postoperative complications.

**Results:**

GLIM-defined malnutrition was diagnosed in143 (71.1%) of the total 201 patients. The median CCI was significantly higher in the GLIM-defined malnutrition group than in the non-malnutrition group (37.2 vs. 28.3; *P* < 0.001). Multivariate logistic regression analysis revealed that GLIM-defined malnutrition (odds ratio 2.87 [95% confidence interval 1.38–5.96], *P* = 0.005) and intraoperative blood loss > 1,000 mL (odds ratio 3.77 [95% confidence interval 1.06–13.47], *P* = 0.041) were independent predictors of high morbidity (CCI ≥ 37.1).

**Conclusion:**

Preoperative GLIM-defined malnutrition was closely associated with increased postoperative complications in patients who underwent resection for BTC.

**Supplementary Information:**

The online version contains supplementary material available at 10.1007/s00595-025-03051-9.

## Introduction

Biliary tract cancer (BTC), including perihilar cholangiocarcinoma (pCCA), distal cholangiocarcinoma (dCCA), ampullary carcinoma (AC), and gallbladder carcinoma (GBC), requires highly invasive surgery, such as pancreaticoduodenectomy (PD) and major hepatectomy with extrahepatic bile duct resection (Hx). Between 2011 and 2020, in-hospital mortality rates for Japanese cohorts decreased from 2.9 to 1.6% for PD and from 3.8 to 2.2% for advanced hepatectomy following the centralization of cases requiring complex surgery to board-certified training institutions and the development of surgical techniques, along with improved perioperative management [1–3]. Nevertheless, the postoperative complications and in-hospital mortality rates after these procedures are higher than those after other procedures [4]. Moreover, PD and Hx for BTC have been reported to have even higher risks of complications and mortality [5–9]. Therefore, it is important for biliary surgeons to identify the risk factors for postoperative complications after these procedures, especially if they can be addressed preoperatively.

In 2018, the Global Leadership Initiative on Malnutrition (GLIM) criteria were proposed to offer universal diagnostic criteria for malnutrition [10]. The components of the GLIM criteria are used worldwide because they are so easy to use. In recent years, malnutrition diagnosed using the GLIM criteria (GLIM-defined malnutrition) has been reported as a prognostic factor for various cancers [11–16] and a risk factor for postoperative complications [11, 12, 14]. Previously, we reported that GLIM-defined severe malnutrition was an independent prognostic factor for the OS (hazard ratio 1.68, 95% confidence interval [CI] 1.06–2.66, *P* = 0.0282) of patients with resected extrahepatic cholangiocarcinoma [15]. However, its clinical relevance as a risk factor for postoperative complications, especially in patients with resected BTC, has not been investigated. This may be due to the high prevalence of severe postoperative complications regardless of the existence of preoperative malnutrition.

The Clavien–Dindo Classification (CDC), which grades postoperative complications according to the invasiveness of intervention for most serious postoperative complications, is a standard tool for assessing the postoperative complications of several areas of surgery because of its simplicity [17, 18]. However, the CDC does not consider accumulated multiple complications and may not adequately describe the postoperative complications of highly invasive procedures that are prone to serious or multiple complications [19]. To address this issue, Slankamenac et al. [20] formulated the comprehensive complication index (CCI), which integrates all the postoperative complications in accordance with the CDC grade. Its value is expressed as a continuous variable ranging from 0 (no complications) to 100 (death). Highly invasive procedures such as PD and Hx are likely to have multiple complications, and assessing complications using the CCI may provide more accurate and objective comprehensive evaluations of patients’ postoperative status than the previously studied approach of dichotomizing complications, for example, CDC ≥ IIIa as major complications and others as minor complications [19, 21].

Based on these points, we used the CCI to investigate the impact of GLIM-defined malnutrition on postoperative complications after resection for BTC.

## Methods

### Study design

Between January 2013 and December 2021, 260 consecutive patients underwent resection for histopathologically proven BTC, at the Department of Hepato-Biliary-Pancreatic Surgery, Kobe University. We analyzed, retrospectively, 201 of these 260 patients who underwent the highly invasive procedures of PD or Hx. Clinical and pathological data were collected from the patients’ medical charts. Data on weight change were obtained by subtracting the weight at their initial visit (excluding patients with data related to other diseases) from the weight taken the day before surgery. Nutritional scores were calculated using blood test data obtained the day before surgery. Pathological findings were evaluated according to the Union for International Cancer Control TNM staging system, 7 th edition [22]. Skeletal muscle mass was assessed by calculating the cross-sectional area (cm^2^) of skeletal muscle mass at the level of the third lumbar vertebra (L3). This calculation was done using plain computed tomography (CT) images that were taken within 2 months before surgery and the Ziostation2 image analysis software (Ziosoft, Tokyo, Japan). Regions with attenuation values of − 29 ± 150 Hounsfield units (HU) were identified and quantified as skeletal muscle areas.

The cross-sectional areas (cm^2^) of skeletal muscle mass at the level of L3 level were normalized as follows: Skeletal muscle index at L3 level (L3-SMI, cm^2^/m^2^) = cross-sectional area of skeletal muscle (cm^2^)/patient height^2^ (m^2^).

### Preoperative management

To plan the treatment strategies, all patients underwent laboratory investigations and imaging, including multi-detector row CT (MDCT), endoscopic retrograde cholangiography, and magnetic resonance cholangiopancreatography. Patients who met the criteria of the Tokyo Guidelines 2018 were diagnosed with preoperative cholangitis [23]. If necessary, preoperative biliary drainage procedures, including endoscopic biliary drainage and percutaneous transhepatic biliary drainage, were performed. The Indocyanine green test was performed when the total bilirubin levels were ≤ 3 mg/dL. In cases requiring Hx with estimated future liver remnant volume of ≤ 40%, portal vein embolization was performed 4–7 weeks prior to the resection.

### Surgery

The indications and type of surgery were based on the intraoperative findings and determinations made by a multidisciplinary team. With regard to PD, subtotal stomach-preserving PD with conventional lymph node dissection was the standard procedure (*n* = 114, 89.0%) [24], while pylorus-preserving PD was performed in 8 patients (6.3%), and conventional PD was performed in 6 (4.7%). Reconstruction was conducted using the modified Child method after resection. We added gallbladder bed resection when PD was performed for GBC. In this study, Hx included right hemihepatectomy, right trisectionectomy, left hemihepatectomy, and left trisectionectomy extending to the caudate lobe with extrahepatic bile duct resection. Biliary tract reconstruction was performed using Roux-en-Y hepaticojejunostomy. Combined major vascular resection and reconstruction (MVR) were performed, such as portal vein and hepatic artery, when radical resection was considered possible. Hepatopancreaticoduodenectomy was not performed in any of the patients in this study. Given the known differences in postoperative complications and patient recovery according to the type of procedure, the study cohort was grouped into two subgroups: PD and Hx, and all analyses were performed for each subgroup.

### Postoperative management

Intensive care management of patients in the acute postoperative period and during the event of critical illness was coordinated by a team of intensivists. To prevent anastomotic ulcers, proton pump inhibitors were administered intravenously on the night of postoperative day (POD) 0 and then switched to oral administration after the patient started oral nutritional intake. The nasogastric tube was removed on POD 1. Oral nutritional intake was generally started on PODs 4–6, although in some cases, this was postponed depending on the clinical presentation, such as abdominal distention or vomiting. Patients were encouraged to mobilize early after surgery. MDCT was performed routinely on around PODs 7–10, mainly to evaluate undrained fluid collection, pseudoaneurysms, and liver perfusion. Intravenous antibiotics, drain repositioning, and additional percutaneous drain placement were considered if there was undrained fluid collection or intra-abdominal abscess. Prophylactic anticoagulants were not administered routinely.

### Evaluation of postoperative complications

Postoperative complications were assessed using the methods reported by Kawakatsu et al. [19, 25]. All postoperative complications were evaluated using the CDC [18] and the CCI [20], which was calculated as the sum of severity of the complications and was the primary outcome of this study. The CCI was calculated using an online calculator (https://www.cci-calculator.com/). High morbidity caused by postoperative complications was defined as a 90-day cumulative CCI ≥ 37.1, which indicates the presence of at least two CDC grade IIIa complications [26, 27]. To calculate the CCI, we re-reviewed the medical charts documented by all the medical staff, laboratory investigations, imaging studies, and drug and blood transfusion orders. Postoperative pancreatic fistula (POPF) and delayed gastric emptying (DGE) were diagnosed according to the International Study Group of Pancreatic Surgery classification [28, 29]. Bile leakage and post-hepatectomy liver failure (PHLF) were diagnosed in accordance with the International Study Group of Liver Surgery classification [30, 31]. Grades B/C for each complication were defined as clinically relevant. Patients with intra-abdominal infected fluid collection were considered to have an intra-abdominal abscess if a new puncture (CDC grade IIIa) or new intravenous antibiotics (CDC grade II) were required. Readmission was defined as hospitalization within 90 days of discharge.

### Definition of GLIM-defined malnutrition in this study

The GLIM criteria involve a two-step approach, wherein the risk screening is followed by a diagnosis of malnutrition [10]. When patients were identified as “at risk” using any validated screening tool and fulfilled at least one phenotypic criterion (non-volitional weight loss, low BMI, or decreased muscle mass) and one etiologic criterion (decreased food intake/assimilation or disease burden/inflammatory status), malnutrition was diagnosed [10]. In this study, risk screening was omitted because of the unavailability of data required for a validated screening tool, and we contemplated that it was acceptable to omit risk screening because participants with BTC in this study were at risk for malnutrition [12, 15]. Additionally, since we considered that patients with BTC already met one etiologic criterion (disease burden), the diagnosis of malnutrition was made if they met any of the phenotypic criterion [15]. The components of each phenotypic criterion are as follows: non-volitional weight loss was defined as > 5% weight loss within the past 6 months or > 10% weight loss beyond 6 months [10]. Low BMI cut-off values were defined as < 18.5 kg/m^2^ for patients aged < 70 years or < 20 kg/m^2^ for those aged ≥ 70 years, based on previous studies in Asian populations [32, 33]. L3-SMI cut-off values were set as the mean – 1 standard deviation (SD) in young Asians [34], 45.0 cm^2^/m^2^ for male patients and 34.0 cm^2^/m^2^ for female patients. Eligible patients were classified into GLIM-defined malnutrition and non-malnutrition groups. To further investigate the association between the GLIM-defined malnutrition severity and the CCI, patients were classified into moderate and severe malnutrition groups based on previous reports [15, 16].

To examine the relationship between other nutritional scores and the CCI, we calculated the controlling nutritional status (CONUT), the Glasgow prognostic score (GPS), the modified Glasgow prognostic score (mGPS), and the prognostic nutritional index (PNI), using cut-off values determined by previous studies [35–39].

### Statistical analysis

Statistical analyses were performed using JMP version 15 software (SAS, Cary, NC, USA). Continuous variables are presented as medians with interquartile ranges (IQRs) and compared using the Mann–Whitney U test. Categorical variables are presented as numeric values with percentages and compared using Pearson’s Chi-square test or Fisher’s exact test. The association between the malnutrition severity (non-malnutrition, moderate malnutrition, and severe malnutrition groups) and the CCI was assessed using the Jonckheere–Terpstra trend test. Univariate analysis was conducted to detect potential risk factors for high morbidity (CCI ≥37.1). Variables that were* P* < 0.100 in the univariate analysis or those that were shown to be risk factors in previous studies were entered into a multivariate logistic regression analysis to identify factors independently associated with high morbidity, and the results are presented as odds ratios (ORs) and 95% CIs. Two-sided *P* < 0.050 was considered to indicate significance.

## Results

### Patient’s characteristics

The study cohort comprised 133 men (66.2%) and 68 women (33.8%), with a median age of 71 years (IQR, 65–76 years). There were 58 patients (28.9%) with dCCA, 63 (31.3%) with AC, 72 (35.8%) with pCCA, and 8 (4.0%) with GBC. One-hundred-and-twenty-eight patients (63.7%) underwent PD, 73 (36.3%) underwent Hx, and 11 (5.5%) underwent combined MVR.

Table 1 shows the comparison of the patient characteristics by nutritional status for the entire cohort. According to the GLIM criteria, 143 patients (71.1%) were classified into the GLIM-defined malnutrition group and the remaining (*n* = 58, 28.9%), into the non-malnutrition group. GLIM-defined malnutrition was significantly associated with older age (*P* = 0.040), a higher rate of preoperative cholangitis (*P* = 0.019), primary tumor site (*P* = 0.037), and surgical procedure (*P* = 0.049). There was no association between GLIM-defined malnutrition and background diseases (all *P* ≥ 0.050) other than preoperative cholangitis.

**Table 1:** Clinical characteristics of patients with different nutritional status

	Overall	Non-malnutrition	GLIM-defined malnutrition	*P* value
	*n* = 201	*n* = 58	*n* = 143	
Baseline characteristics				
Age (years)	71 (65–76)	71 (62–75)	72 (67–76)	0.040*
Male gender	133 (66.2%)	36 (62.1%)	97 (67.8%)	0.434
ASA-PS ≥ 3	17 (8.5%)	4 (6.9%)	13 (9.1%)	0.612
Diabetes mellitus	39 (19.4%)	10 (17.2%)	29 (20.4%)	0.606
Pulmonary disease	32 (15.9%)	9 (15.5%)	23 (16.1%)	0.920
Cardiovascular disease	49 (24.4%)	10 (17.2%)	39 (27.3%)	0.133
Chronic kidney disease	41 (20.4%)	12 (20.7%)	29 (20.3%)	0.948
Hypertension	105 (52.2%)	30 (51.7%)	75 (52.5%)	0.926
Dyslipidemia	57 (28.4%)	21 (36.2%)	36 (25.2%)	0.116
Antithrombotic therapy	39 (19.4%)	10 (17.2%)	29 (20.3%)	0.622
Preoperative cholangitis	81 (40.3%)	16 (27.6%)	65 (45.5%)	0.019*
Primary tumor site				0.037*
Distal	58 (28.9%)	17 (29.3%)	41 (28.7%)	
Ampullary	63 (31.3%)	24 (41.4%)	39 (27.3%)	
Perihilar	72 (35.8%)	13 (22.4%)	59 (41.3%)	
Gallbladder	8 (4.0%)	4 (6.9%)	4 (2.7%)	
UICC 7 th pT3-4	77 (38.3%)	17 (29.3%)	60 (42.0%)	0.095
Lymph node metastasis	71 (35.3%)	18 (31.0%)	53 (37.1%)	0.418
Preoperative chemotherapy	6 (3.0%)	1 (1.7%)	5 (3.5%)	0.504
Operative characteristics				
Surgical procedure				0.049*
PD	128 (63.7%)	43 (74.1%)	85 (59.4%)	
Hx	73 (36.3%)	15 (25.9%)	58 (40.6%)	
Operation time (min)	517 (443–579)	509 (441–572)	523 (445–588)	0.394
Blood loss (ml)	420 (233–648)	385 (200–566)	450 (250–660)	0.262
Combined MVR	11 (5.5%)	1 (1.7%)	10 (7.0%)	0.137

Values represent the number of patients (%), or median (interquartile range)

*GLIM* global leadership initiative on malnutrition, *ASA-PS* American Society of Anesthesiologists physical status, *UICC* Union for International Cancer Control, *PD* pancreaticoduodenectomy, *Hx* major hepatectomy with extrahepatic bile duct resection, *MVR* major vascular resection and reconstruction

^*^P < 0.050

### Postoperative outcomes

**Fig. 1:**
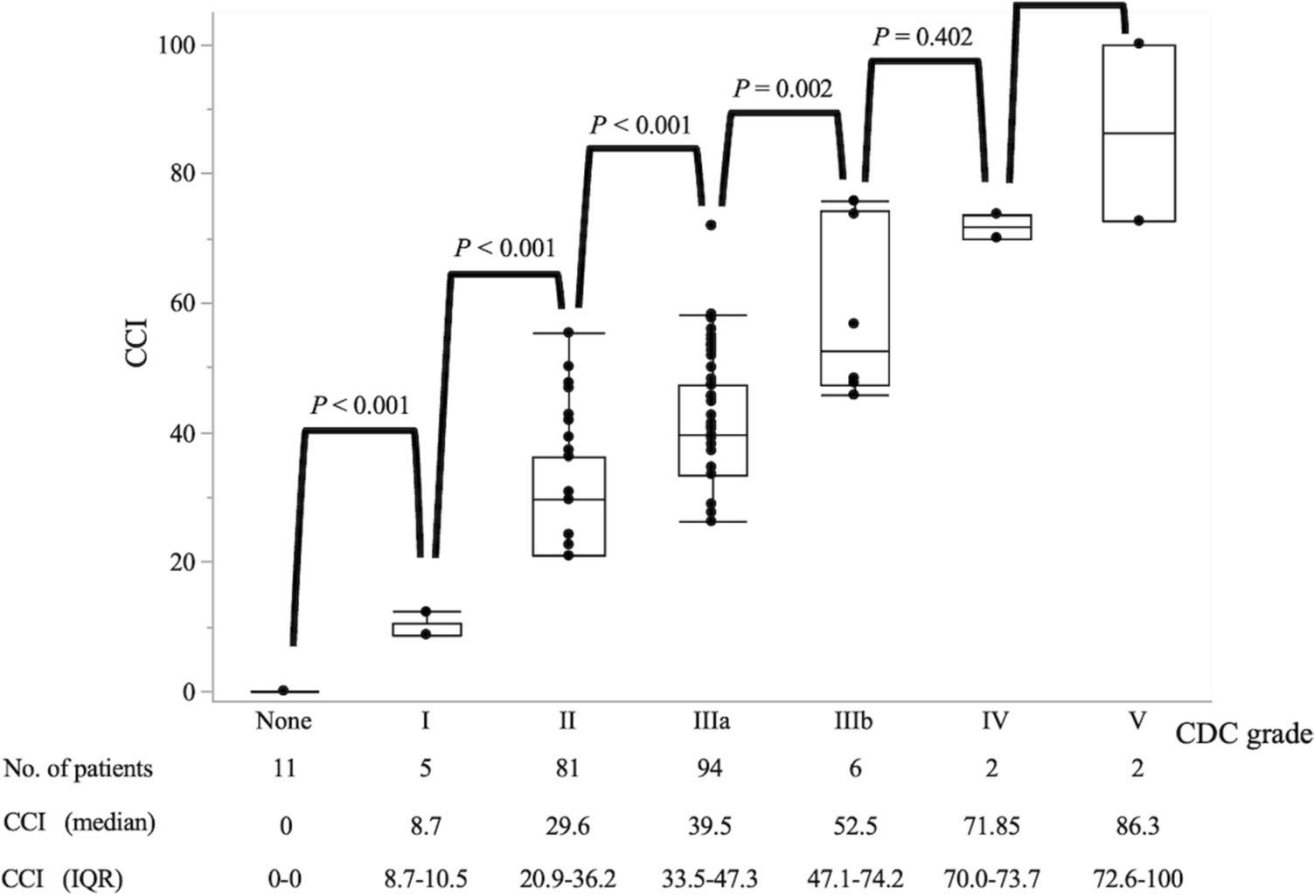
Associations between the highest Clavien–Dindo classification grade during the hospital stay and the comprehensive complication index. Box-and-whisker plots display median, interquartile range, and range. *CDC* Clavien–Dindo classification, *CCI* comprehensive complication index, *IQR* interquartile range

Table 2 shows the comparison of the postoperative outcomes of patients with a CCI < 37.1 and those with a CCI ≥ 37.1. A CCI ≥ 37.1 was associated with significantly prolonged postoperative hospital stay (*P* < 0.001), more readmissions (*P* = 0.002), more reoperations (*P* < 0.001), and more IVRs for postoperative bleeding (*P* < 0.001). As Fig. 1 shows, the highest CDC grade of complications that occurred during the hospital stay was no complications in 11 (5.5%), grade I in 5 (2.5%), grade II in 81 (40.3%), grade IIIa in 94 (46.7%), grade IIIb in 6 (3.0%), grade IV in 2 (1.0%), and grade V in 2 (1.0%) patients. The median CCI score increased with increasing CDC grade among CDC grades I–IIIb (*P* < 0.001).

**Table 2:** Relationship between the comprehensive complication index and postoperative outcomes

	CCI < 37.1	CCI ≥ 37.1	*P* value
	*n* = 116	*n* = 85	
Postoperative hospital stay (days)	26 (19–38)	48 (34–72)	< 0.001*
Readmission	12 (10.3%)	23 (27.1%)	0.002*
Reoperation	0 (0%)	9 (10.6%)	< 0.001*
IVR for bleeding	0 (0%)	10 (11.8%)	< 0.001*
90 days-mortality	1 (0.9%)	1 (1.2%)	0.824

Values are number of patients (%), or median (interquartile range)

*CCI* comprehensive complication index, *IVR* interventional radiology

^*^P < 0.050

Table 3 shows the comparison of the postoperative outcomes according to the nutritional status for the entire cohort. CCI was higher in the GLIM-defined malnutrition group (37.2 vs. 28.3, *P* < 0.001), while the rate of CDC grade ≥ IIIa was not significantly different (*P* = 0.352). When comparing the CCI by malnutrition severity (non-malnutrition [*n* = 58, 28.9%] vs. moderate malnutrition [*n* = 93, 46.2%] vs. severe malnutrition groups [*n* = 50, 24.9%]), the CCI values were 28.3 (20.9–36.2) vs. 36.2 (28.6–45.1) vs. 39.5 (29.1–46.1), respectively. There were significant differences between the non-malnutrition and moderate malnutrition groups (*P* = 0.005) and between the non-malnutrition and severe malnutrition groups (*P* = 0.002), but not between the moderate and severe malnutrition groups (*P* = 0.407). Trend analysis using the Jonckheere–Terpstra trend test showed a significant increase in the CCI with increasing malnutrition severity (*P*-for-trend < 0.001). Moreover, we analyzed the association between the CCI and various nutritional scores, including CONUT, GPS, mGPS, and PNI. Among these, high CONUT (≥ 5), high GPS (≥ 1), and low PNI (< 45) were significantly associated with the increased CCI (all *P* < 0.001) (Table S1).

**Table 3:** Postoperative outcomes of patients with different nutritional status

	Overall	Non-malnutrition	GLIM-defined malnutrition	*P* value
	*n* = 201	*n* = 58	*n* = 143	
Postoperative outcomes				
CCI	34.6 (26.2–42.6)	28.3 (20.9–36.2)	37.2 (29.6–45.4)	< 0.001*
CDC ≥ IIIa	104 (51.7%)	33 (56.9%)	71 (49.7%)	0.352
Postoperative hospital stay (days)	32 (22–48)	32 (24–42)	35 (22–51)	0.471
Readmission	35 (17.4%)	9 (15.5%)	26 (18.2%)	0.652
Reoperation	9 (4.5%)	1 (1.7%)	8 (5.6%)	0.229
IVR for bleeding	10 (5.0%)	3 (5.2%)	7 (4.9%)	0.935
90 days-mortality	2 (1.0%)	1 (1.7%)	1 (0.7%)	0.507

Values represent the number of patients (%), or median (interquartile range)

*GLIM* global leadership initiative on malnutrition, *CCI* comprehensive complication index, *CDC* Clavien-Dindo classification, *IVR* interventional radiology

^*^P < 0.050

When comparing postoperative outcomes by type of surgery in patients who underwent PD, the CCI was higher in the GLIM-defined malnutrition group (34.6 vs. 27.6, *P* = 0.002). In contrast, the difference in CCI between the GLIM-defined malnutrition and non-malnutrition groups in patients who underwent Hx was not significant (39.5 vs. 33.5, *P* = 0.200) (Table S2).

### Univariate and multivariate analyses of factors associated with high morbidity (CCI ≥ 37.1)

Table 4 shows the factors associated with high morbidity (CCI ≥ 37.1) in the entire cohort (*n* = 201). Eighty-five patients had a CCI ≥ 37.1. Univariate analysis revealed that age ≥ 70 years, preoperative cholangitis, GLIM-defined malnutrition, operation time > 600 min, intraoperative blood loss > 1000 mL, and surgical procedure (Hx) were potential risk factors associated with high morbidity (all *P* < 0.100). Multivariate analysis revealed that GLIM-defined malnutrition (OR 2.87, 95% CI 1.38–5.96, *P* = 0.005) and intraoperative blood loss > 1000 mL (OR 3.77, 95% CI 1.06–13.47, *P* = 0.041) were independent predictors of high morbidity. Multivariate analyses incorporating each nutritional score (CONUT, GPS, mGPS, and PNI) as covariates, along with age, preoperative cholangitis, operation time, intraoperative blood loss, and surgical procedure (using the same covariates as in Table 4) to identify risk factors for high morbidity (CCI ≥ 37.1), showed that none of these scores remained independent risk factors (all *P* > 0.050) (Table S3).

**Table 4:** Univariate and multivariate analyses of the risk factors for high morbidity (CCI ≥ 37.1) in patients undergoing curative-intent resection for biliary tract cancer

	*n*	Univariate		Multivariate	
		*P* value	OR (95% CI)	*P* value	OR (95% CI)
High morbidity (CCI ≥ 37.1)					
Sex (male/female)	133/68	0.258	1.41 (0.78–2.58)		
Age (≥ 70/< 70 years)	118/83	0.078	1.68 (0.94–3.00)	0.057	1.84 (0.98–3.47)
ASA-PS (≥ 3/< 3)	17/184	0.678	1.24 (0.46–3.35)		
Diabetes mellitus (+/-)	39/162	0.859	0.94 (0.46–1.91)		
Pulmonary disease (+/-)	32/169	0.836	0.92 (0.43–1.99)		
Cardiovascular disease (+/-)	49/152	0.449	1.28 (0.67–2.45)		
Chronic kidney disease (+/-)	41/160	0.347	1.39 (0.70–2.77)		
Hypertension (+/-)	105/96	0.190	1.46 (0.83–2.56)		
Dyslipidemia (+/-)	57/144	0.974	0.99 (0.53–1.84)		
Antithrombotic therapy (+/-)	39/162	0.587	1.22 (0.60–2.45)		
Preoperative cholangitis (+/-)	81/120	0.025*	1.93 (1.09–3.42)	0.060	1.81 (0.98–3.38)
GLIM-defined malnutrition (+/-)	143/58	< 0.001*	3.51 (1.75–7.06)	0.005*	2.87 (1.38–5.96)
Preoperative chemotherapy (+/-)	6/195	0.699	1.38 (0.27–7.00)		
Operation time (> 600/≤ 600 min)	40/161	0.032*	2.16 (1.07–4.36)	0.338	1.49 (0.66–3.39)
Intraoperative blood loss (> 1000/≤ 1000 mL)	15/186	0.018*	4.16 (1.28–13.56)	0.041*	3.77 (1.06–13.47)
Surgical procedure (Hx/PD)	73/128	0.035*	1.87 (1.04–3.35)	0.141	1.65 (0.85–3.19)
Combined MVR (+/-)	11/190	0.152	2.51 (0.71–8.88)		
UICC 7 th pT (3–4/1–2)	77/124	0.313	1.34 (0.76–2.39)		
Lymph node metastasis (+/-)	71/130	0.236	1.42 (0.79–2.55)		

*CCI* comprehensive complication index, *OR* odds ratio, *CI* confidence interval, *ASA-PS* American Society of Anesthesiologists physical status, *GLIM* global leadership initiative on malnutrition, *Hx* major hepatectomy with extrahepatic bile duct resection, *PD* pancreaticoduodenectomy, *MVR* major vascular resection and reconstruction, *UICC* Union for International Cancer Control

^*^P < 0.050

When a multivariate analysis of risk factors for high morbidity was performed for each surgical procedure, only GLIM-defined malnutrition (OR 4.01, 95% CI 1.50–10.76, *P* = 0.006) was found to be an independent predictor of high morbidity in patients who underwent PD. Conversely, no independent risk factors for high morbidity were identified in patients who underwent Hx (Table S4).

## Discussion

We found that patients with preoperative GLIM-defined malnutrition had a higher CCI after resection for BTC. Furthermore, multivariate analysis identified that preoperative GLIM-defined malnutrition and intraoperative blood loss > 1000 mL were independent risk factors for high morbidity (CCI ≥ 37.1) in patients who underwent resection for BTC. In patients who underwent PD, GLIM-defined malnutrition was independently associated with more postoperative complications, whereas this association was not observed in those who underwent Hx. To our knowledge, this is the first study to show an association between GLIM-defined malnutrition and postoperative morbidity, evaluated with the CCI, in patients who underwent resection for BTC.

The CCI is considered more sensitive than the CDC because it is calculated by aggregating all the postoperative complications according to severity [20, 21]. Therefore, the CCI may be a better indicator of complications than the CDC in groups at high risk of multiple postoperative complications, such as patients undergoing resection for BTC [25]. Previous studies have shown that a higher CCI is associated with higher overall postoperative costs [40, 41], a longer postoperative hospital stay [25, 41], and poorer cancer-specific survival [42]. Previous studies have found that the CCI correlates more strongly with the postoperative hospital stay [43, 44] and postoperative costs [43] than the CDC in pancreatectomy cohorts. Moreover, in a pCCA cohort, classification based on the early postoperative CCI has been shown to accurately predict the trajectory of serious postoperative outcomes [19]. In this study, we found that a CCI ≥ 37.1 was associated with a prolonged postoperative stay, more readmissions, more reoperations, and more IVRs for postoperative bleeding. Therefore, it seems reasonable to set a CCI ≥ 37.1 as the primary endpoint.

The GLIM criteria were proposed as universal diagnostic criteria to enable a worldwide discussion on the prevalence of malnutrition and optimal interventions and outcomes [10]. Matsui et al. [14] reported in their review that the prevalence of GLIM-defined malnutrition ranged between 11.9 and 88.0% in patients with various cancers. These variations can be attributed to the differences in the combination of criteria and respective cut-off values used to diagnose malnutrition according to the GLIM criteria and differences in patient subgroups. In this study, 71.1% patients with BTC were diagnosed with malnutrition based on the GLIM criteria. In patients with BTC, the high prevalence of preoperative cholangitis and the progressive nature of tumor pathogenesis may account for the high incidence of malnutrition.

GLIM-defined malnutrition is reported to be associated with increased postoperative complications in surgical patients with various cancer types. Matsui et al. [14] performed a systematic review and meta-analysis and concluded that postoperative complications may increase in cancer patients with GLIM-defined malnutrition. Brown et al. [11] performed a systematic review and elicited that GLIM-defined malnutrition in patients with cancer was consistently associated with more postoperative complications and a prolonged postoperative hospital stay. In the field of hepato-biliary-pancreatic (HBP) surgery, an association between GLIM-defined malnutrition and postoperative complications was reported only in a cohort of patients undergoing liver resection for hepatocellular carcinoma [16] and in a cohort of patients undergoing PD for pancreatic ductal adenocarcinoma (PDAC) [13]. However, none of the previous studies included cohorts confined to BTC, a group at higher risk for complications and mortality. The number of surgeries for BTC is small within the total number of HBP surgeries, but it is not insignificant [45]. The present study builds on these other studies and enhances them by demonstrating the ability of the GLIM criteria to predict the short-term outcomes of patients undergoing resection for BTC.

The results of our multivariate analysis showed that preoperative GLIM-defined malnutrition and intraoperative blood loss > 1000 mL were independent risk factors for high morbidity (CCI ≥ 37.1). This suggests that the preoperative nutritional status is a risk factor for postoperative complications as well as for intraoperative blood loss, an indicator of the invasiveness of surgery. The most important component of the GLIM criteria underlying this finding may be decreased muscle mass. Loss of muscle mass has been reported as a risk factor for postoperative complications in patients undergoing PD [46, 47] and Hx [48, 49]. Several studies have reported that low BMI [50, 51] and unintentional weight loss [52, 53] are also associated with postoperative complications. In the GLIM criteria, malnutrition is diagnosed if any one of decreased muscle mass, low BMI, or unintentional weight loss is identified. Using this approach, the GLIM criteria outperformed other nutritional scores (CONUT, GPS, mGPS, and PNI) for identifying risk factors for complications in our cohort. As for biological mechanisms, it has been reported that the malnutrition status of patients with sarcopenia may adversely affect postoperative recovery, especially tissue repair and immunological response [54, 55].

In this study, GLIM-defined malnutrition was independently associated with more postoperative complications in patients who underwent PD, whereas no such association was observed in those who underwent Hx. We attribute this to the fact that Hx is a more invasive procedure than PD, making the impact of malnutrition on postoperative complications less apparent. Lee et al. [13] investigated the impact of GLIM-defined malnutrition on short-term outcomes in PDAC patients undergoing PD, reporting a significantly higher overall complication rate (CDC grade ≥ I) in the GLIM-defined malnutrition group than in the non-malnutrition group (44.0% vs. 37.9%, *P* = 0.046). These findings suggest that the impact of GLIM-defined malnutrition on postoperative complications may be specific to PD. However, differences between BTC and PDAC, such as the prevalence of preoperative cholangitis and pancreatic hardness, affect postoperative complications, making it difficult to conclude that the impact of GLIM-defined malnutrition is specific to PD.

Multivariate analysis revealed that intraoperative blood loss > 1,000 mL was also an independent risk factor for high morbidity (CCI ≥ 37.1). Consistent with our result, several previous studies have demonstrated that increased blood loss is associated with poor postoperative outcomes for patients who underwent PD [56] and Hx [57]. Although intraoperative blood loss may be related to tumor aggressiveness to some extent, surgeons should be aware that it is potentially advantageous to reduce intraoperative blood loss to minimize postoperative complications. To improve the outcome of patients undergoing resection for BTC, preoperative nutritional intervention and rehabilitation may be important. However, there is no robust evidence from prospective studies with nutritional intervention and rehabilitation. Yokoyama et al. [58] suggested that interventions such as preoperative exercise and nutritional support for patients with BTC can improve the nutritional status of patients with HBP cancer. We believe that preoperative nutritional intervention and rehabilitation are necessary to improve the postoperative outcomes of patients undergoing surgery for BTC. Further studies are needed to identify the optimal preoperative rehabilitation and nutritional therapy for patients with preoperative GLIM-defined malnutrition. 

This study had some limitations that need consideration. First, it was a single-center retrospective study, and therefore, unintended biases could not be completely excluded. Second, the CCI was the primary outcome in this study. The definition of each postoperative complication is sometimes less objective, depending on differences in institutional postoperative management policies and the manner in which each attending surgeon records them in medical charts. Therefore, caution should be exercised when extrapolating the results of this study, and future prospective studies are needed to ensure the accuracy of the information about postoperative complications. Third, the study cohort included patients with BTC who underwent two major surgical procedures. Although this is representative of a cohort managed in clinical practice, more cases are needed for analysis based on the type of surgery.

## Conclusions

This study provided new evidence that preoperative GLIM-defined malnutrition was closely associated with increased postoperative complications evaluated with the CCI in patients with BTC who underwent resection. This new finding may have important implications for identifying groups that require nutritional intervention and rehabilitation before major surgery commonly associated with postoperative complications.

## Supplementary Information

Below is the link to the electronic supplementary material.

Supplementary file1 (DOCX 32 KB)

Supplementary file2 (DOCX 33 KB)

Supplementary file3 (DOCX 31 KB)

Supplementary file4 (DOCX 42 KB)
