# Supplementary material for: Using the comprehensive complication index to assess the impact of Global Leadership Initiative on Malnutrition (GLIM)-defined malnutrition on postoperative complications after resection for biliary tract cancer
Source: Surg Today. 2025 May 27;55(11):1598–608. doi: 10.1007/s00595-025-03051-9 (PMC12534308; doi:10.1007/s00595-025-03051-9)
Supplement: Supplementary file 1 — Supplementary file1 (DOCX 32 KB) [file 595_2025_3051_MOESM1_ESM.docx]

| **Supplementary Table S1.** Comparison of the comprehensive complication index by the nutritional score categories | | | |
| --- | --- | --- | --- |
| Nutritional score | Category | CCI | *P* value |
| CONUT | High (≥5) | 39.5 (29.6-52.0) | **<0.001*** |
|  | Low (<4) | 33.5 (22.6-41.4) |  |
| GPS | High (≥1) | 39.5 (29.6-50.1) | **<0.001*** |
|  | Low (0) | 29.6 (20.9-39.5) |  |
| mGPS | High (≥1) | 36.2 (28.1-48.1) | 0.255 |
|  | Low (0) | 33.5 (24.2-41.8) |  |
| PNI | Low (<45) | 36.2 (28.3-46.5) | **0.002*** |
|  | High (≥45) | 31.6 (20.9-39.5) |  |
| Values are expressed as the median (interquartile range). *P* <0.050*  *CCI* comprehensive complication index, *CONUT* controlling nutritional status, *GPS* Glasgow prognostic score, *mGPS* modified Glasgow prognostic score, *PNI* prognostic nutritional index. | | | |
