# Supplementary material for: Using the comprehensive complication index to assess the impact of Global Leadership Initiative on Malnutrition (GLIM)-defined malnutrition on postoperative complications after resection for biliary tract cancer
Source: Surg Today. 2025 May 27;55(11):1598–608. doi: 10.1007/s00595-025-03051-9 (PMC12534308; doi:10.1007/s00595-025-03051-9)
Supplement: Supplementary file 2 — Supplementary file2 (DOCX 33 KB) [file 595_2025_3051_MOESM2_ESM.docx]

| **Supplementary Table S2.** Patients’ characteristics and postoperative outcomes classified by surgical procedure (pancreaticoduodenectomy or major hepatectomy with extrahepatic bile duct resection) | | | | | | | | | |
| --- | --- | --- | --- | --- | --- | --- | --- | --- | --- |
|  | PD | | | |  | Hx | | | |
|  | Overall | Non-malnutrition | GLIM-defined malnutrition | *P* value |  | Overall | Non-malnutrition | GLIM-defined malnutrition | *P* value |
|  | *n* = 128 | *n* = 43 | *n* = 85 |  |  | *n* = 73 | *n* = 15 | *n* = 58 |  |
| Baseline characteristics |  |  |  |  |  |  |  |  |  |
| Age (years) | 72 (65-76) | 71 (62-75) | 72 (68-78) | **0.021*** |  | 71 (66-74) | 70 (63-74) | 71 (66-74) | 0.657 |
| Male gender | 85 (66.4%) | 24 (55.8%) | 61 (71.8%) | 0.071 |  | 48 (65.8%) | 12 (80.0%) | 36 (62.1%) | 0.192 |
| ASA-PS ≥3 | 10 (7.8%) | 4 (9.3%) | 6 (7.1%) | 0.655 |  | 7 (9.6%) | 0 (0%) | 7 (12.1%) | 0.157 |
| Diabetes mellitus | 29 (22.7%) | 8 (18.6%) | 21 (24.7%) | 0.436 |  | 10 (13.7%) | 2 (13.3%) | 8 (14.0%) | 0.944 |
| Pulmonary disease | 21 (16.4%) | 7 (16.3%) | 14 (16.5%) | 0.978 |  | 11 (15.1%) | 2 (13.3%) | 9 (15.5%) | 0.833 |
| Cardiovascular disease | 29 (22.7%) | 6 (14.0%) | 23 (27.1%) | 0.094 |  | 20 (27.4%) | 4 (26.7%) | 16 (27.6%) | 0.943 |
| Chronic kidney disease | 22 (17.2%) | 8 (18.6%) | 14 (16.5%) | 0.762 |  | 19 (26.0%) | 4 (26.7%) | 15 (25.9%) | 0.950 |
| Hypertension | 65 (50.8%) | 19 (44.2%) | 46 (54.1%) | 0.288 |  | 40 (54.8%) | 11 (73.3%) | 29 (50.0%) | 0.106 |
| Dyslipidemia | 39 (30.5%) | 15 (34.9%) | 24 (28.2%) | 0.440 |  | 18 (24.7%) | 6 (40.0%) | 12 (20.7%) | 0.122 |
| Antithrombotic therapy | 23 (18.0%) | 6 (14.0%) | 17 (20.0%) | 0.400 |  | 16 (21.9%) | 4 (26.7%) | 12 (20.7%) | 0.618 |
| Preoperative cholangitis | 51 (39.8%) | 10 (23.3%) | 41 (48.2%) | **0.006*** |  | 30 (41.1%) | 6 (40.0%) | 24 (41.4%) | 0.923 |
| Primary tumor site |  |  |  | 0.757 |  |  |  |  | **0.024*** |
| Distal | 58 (45.3%) | 17 (39.6%) | 41 (48.3%) |  |  | 0 (0%) | 0 (0%) | 0 (0%) |  |
| Ampullary | 63 (49.2%) | 24 (55.8%) | 39 (45.9%) |  |  | 0 (0%) | 0 (0%) | 0 (0%) |  |
| Perihilar | 4 (3.1%) | 1 (2.3%) | 3 (3.5%) |  |  | 68 (93.2%) | 12 (80%) | 56 (96.6%) |  |
| Gallbladder | 3 (2.4%) | 1 (2.3%) | 2 (2.3%) |  |  | 5 (6.8%) | 3 (20%) | 2 (3.4%) |  |
| MPD diameter (mm) | 3 (2-3) | 2 (2-3) | 3 (2-3.8) | 0.200 |  | - | - | - |  |
| Soft pancreas | 111 (86.7%) | 40 (93.0%) | 71 (83.5%) | 0.135 |  | - | - | - |  |
| ICG-Krem | - | - | **-** |  |  | 0.087 (0.064-0.118) | 0.106 (0.075-0.124) | 0.083 (0.061-0.116) | 0.085 |
| Preoperative PVE | - | - | **-** |  |  | 15 (20.6%) | 2 (13.3%) | 13 (22.4%) | 0.438 |
| Right sided hepatectomy | - | - | **-** |  |  | 35 (47.9%) | 6 (40.0%) | 29 (50.0%) | 0.490 |
| UICC 7^th^ pT3-4 | 51 (39.8%) | 10 (23.3%) | 41 (48.2%) | **0.006*** |  | 26 (35.6%) | 7 (46.7%) | 19 (32.8%) | 0.316 |
| Lymph node metastasis | 47 (36.7%) | 14 (32.6%) | 33 (38.8%) | 0.487 |  | 24 (32.9%) | 4 (26.7%) | 20 (34.5%) | 0.566 |
| Preoperative chemotherapy | 1 (0.8%) | 0 (0%) | 1 (1.2%) | 0.475 |  | 5 (6.9%) | 1 (6.7%) | 4 (6.9%) | 0.975 |
| Operative characteristics |  |  |  |  |  |  |  |  |  |
| Operation time (min) | 475 (420-548) | 476 (426-544) | 474 (416-549) | 0.749 |  | 575 (518-633) | 585 (517-657) | 573 (523-619) | 0.702 |
| Blood loss (ml) | 383 (185-607) | 350 (180-551) | 420 (190-620) | 0.371 |  | 470 (305-738) | 480 (340-743) | 465 (298-740) | 0.999 |
| Combined MVR | 2 (1.6%) | 0 (0%) | 2 (2.4%) | 0.311 |  | 9 (12.3%) | 1 (6.7%) | 8 (13.8%) | 0.454 |
| Postoperative outcomes |  |  |  |  |  |  |  |  |  |
| CR-POPF | 58 (45.3%) | 21 (48.8%) | 37 (43.5%) | 0.569 |  | 1 (1.4%) | 0 (0%) | 1 (1.7%) | 0.609 |
| Intraabdominal hemorrhage | 8 (6.3%) | 3 (7.0%) | 5 (5.9%) | 0.809 |  | 2 (2.7%) | 1 (6.7%) | 1 (1.7%) | 0.296 |
| CR-PHLF | 0 (0%) | 0 (0%) | 0 (0%) | - |  | 17 (23.3%) | 2 (13.3%) | 15 (25.9%) | 0.306 |
| CR-BL | 1 (0.8%) | 1 (2.3%) | 0 (0%) | 0.158 |  | 18 (24.7%) | 5 (33.3%) | 13 (22.4%) | 0.382 |
| DGE | 12 (9.4%) | 2 (4.7%) | 10 (11.8%) | 0.192 |  | 4 (5.5%) | 0 (0%) | 4 (6.9%) | 0.296 |
| CCI | 33.5 (26.2-40.3) | 27.6 (20.9-33.5) | 34.6 (26.2-43.7) | **0.002*** |  | 37.2 (26.1-47.4) | 33.5 (20.9-47.4) | 39.5 (29.6-48.8) | 0.200 |
| CDC ≥IIIa | 75 (58.6%) | 27 (62.8%) | 48 (56.5%) | 0.493 |  | 29 (39.7%) | 6 (40.0%) | 23 (39.7%) | 0.981 |
| Postoperative hospital stay (days) | 32 (22-45) | 32 (25-42) | 31 (22-50) | 0.878 |  | 35 (22-58) | 29 (19-64) | 37 (23-58) | 0.535 |
| Readmission | 20 (15.6%) | 6 (14.0%) | 14 (16.5%) | 0.711 |  | 15 (20.5%) | 3 (20.0%) | 12 (20.7%) | 0.953 |
| Reoperation | 4 (3.1%) | 0 (0%) | 4 (4.7%) | 0.148 |  | 5 (6.8%) | 1 (6.7%) | 4 (6.9%) | 0.975 |
| IVR for bleeding | 9 (7.0%) | 3 (7.0%) | 6 (7.1%) | 0.986 |  | 1 (1.4%) | 0 (0%) | 1 (1.7%) | 0.609 |
| 90 days-mortality | 1 (0.8%) | 1 (2.3%) | 0 (0%) | 0.158 |  | 1 (1.4%) | 1 (6.7%) | 0 (0%) | 0.073 |
| Values represent the number of patients (%), or median (interquartile range). *P* <0.050*  *GLIM* global leadership initiative on malnutrition, *PD* pancreaticoduodenectomy, *Hx* major hepatectomy with extrahepatic bile duct resection, *ASA-PS* American Society of Anesthesiologists physical status, *MPD* main pancreatic duct, *ICG-Krem* indocyanine green clearance of remnant liver, *PVE* portal vein embolization, *UICC* Union for International Cancer Control, *MVR* major vascular resection and reconstruction, *CR-POPF* clinically relevant postoperative pancreatic fistula, *CR-PHLF* clinically relevant post-hepatectomy liver failure, *CR-BL* clinically relevant bile leakage, *DGE* delayed gastric emptying, *CCI* comprehensive complication index, *CDC* Clavien-Dindo classification, *IVR* interventional radiology. | | | | | | | | | |
