# Supplementary material for: Using the comprehensive complication index to assess the impact of Global Leadership Initiative on Malnutrition (GLIM)-defined malnutrition on postoperative complications after resection for biliary tract cancer
Source: Surg Today. 2025 May 27;55(11):1598–608. doi: 10.1007/s00595-025-03051-9 (PMC12534308; doi:10.1007/s00595-025-03051-9)
Supplement: Supplementary file 3 — Supplementary file3 (DOCX 31 KB) [file 595_2025_3051_MOESM3_ESM.docx]

| **Supplementary Table S3.** Multivariate analyses of the risk factors for high morbidity (CCI ≥37.1), with a focus on nutritional scores in patients undergoing curative-intent resection for biliary tract cancer | | | |
| --- | --- | --- | --- |
| High morbidity (CCI ≥37.1) | | | |
|  | *n* | OR (95% CI) | *P* value |
| CONUT (High/Low) | 50/151 | 1.51 (0.74-3.11) | 0.257 |
| GPS (High/Low) | 86/115 | 1.61 (0.84-3.07) | 0.147 |
| mGPS (High/Low) | 32/169 | 0.95 (0.41-2.13) | 0.895 |
| PNI (Low/High) | 113/88 | 1.38 (0.73-2.61) | 0.327 |
| Each score was analyzed separately in a multivariate model adjusting for age, preoperative cholangitis, operation time, intraoperative blood loss, and surgical procedure. No scores remained independent risk factors for high morbidity (CCI ≥37.1).  *CCI* comprehensive complication index, *OR* odds ratio, *CI* confidence interval, *CONUT* controlling nutritional status, *GPS* Glasgow prognostic score, *mGPS* modified Glasgow prognostic score, *PNI* prognostic nutritional index. | | | |
