# Supplementary material for: Using the comprehensive complication index to assess the impact of Global Leadership Initiative on Malnutrition (GLIM)-defined malnutrition on postoperative complications after resection for biliary tract cancer
Source: Surg Today. 2025 May 27;55(11):1598–608. doi: 10.1007/s00595-025-03051-9 (PMC12534308; doi:10.1007/s00595-025-03051-9)
Supplement: Supplementary file 4 — Supplementary file4 (DOCX 42 KB) [file 595_2025_3051_MOESM4_ESM.docx]

| **Supplementary Table S4.** Univariate and multivariate analyses of the risk factors for high morbidity (CCI ≥37.1) classified by surgical procedure (pancreaticoduodenectomy or major hepatectomy with extrahepatic bile duct resection) | | | | | | | | | | | | | |
| --- | --- | --- | --- | --- | --- | --- | --- | --- | --- | --- | --- | --- | --- |
| High morbidity (CCI ≥37.1) | | | | | | | | | | | | | |
|  | PD (*n* = 128) | | | | | |  | Hx (*n* = 73) | | | | | |
|  | *n* | Univariate | |  | Multivariate | |  | *n* | Univariate | |  | Multivariate | |
|  |  | *P* value | OR (95% CI) |  | *P* value | OR (95% CI) |  |  | *P* value | OR (95% CI) |  | *P* value | OR (95% CI) |
| Sex (male/female) | 85/43 | 0.144 | 1.81 (0.82-4.00) |  |  |  |  | 48/25 | 0.995 | 1.00 (0.38-2.64) |  |  |  |
| Age (≥70/<70 years) | 79/49 | **0.026*** | 2.45 (1.12-5.39) |  | 0.065 | 2.23 (0.95-5.26) |  | 39/34 | 0.743 | 1.17 (0.46-2.93) |  |  |  |
| ASA-PS (≥3/<3) | 10/118 | 0.647 | 0.72 (0.18-2.93) |  |  |  |  | 7/66 | 0.293 | 2.50 (0.45-13.81) |  |  |  |
| Diabetes mellitus (+/-) | 29/99 | 0.777 | 0.88 (0.37-2.10) |  |  |  |  | 10/63 | 0.590 | 1.45 (0.37-5.65) |  |  |  |
| Pulmonary disease (+/-) | 21/107 | 0.527 | 1.36 (0.53-3.52) |  |  |  |  | 11/62 | 0.265 | 0.47 (0.12-1.77) |  |  |  |
| Cardiovascular disease (+/-) | 29/99 | 0.554 | 1.29 (0.55-3.01) |  |  |  |  | 20/53 | 0.757 | 1.18 (0.42-3.31) |  |  |  |
| Chronic kidney disease (+/-) | 22/106 | 0.353 | 1.55 (0.61-3.94) |  |  |  |  | 19/54 | 0.953 | 1.03 (0.36-2.94) |  |  |  |
| Hypertension (+/-) | 65/63 | 0.252 | 1.53 (0.74-3.15) |  |  |  |  | 40/33 | 0.580 | 1.30 ()0.52-3.27 |  |  |  |
| Dyslipidemia (+/-) | 39/89 | 0.599 | 0.81 (0.37-1.78) |  |  |  |  | 18/55 | 0.378 | 1.63 (0.55-4.82) |  |  |  |
| Antithrombotic therapy (+/-) | 23/105 | 0.791 | 1.13 (0.45-2.86) |  |  |  |  | 16/57 | 0.704 | 1.24 (0.41-3.79) |  |  |  |
| Preoperative cholangitis (+/-) | 51/77 | **0.049*** | 2.09 (1.00-4.35) |  | 0.186 | 1.75 (0.76-4.01) |  | 30/43 | 0.258 | 1.725 (0.67-4.44) |  |  |  |
| GLIM-defined malnutrition (+/-) | 85/43 | **0.001*** | 4.57 (1.83-11.41) |  | **0.006*** | 4.01 (1.50-10.76) |  | 58/15 | 0.298 | 1.85 (0.58-5.86) |  | 0.394 | 1.70 (0.50-5.78) |
| Preoperative chemotherapy (+/-) | 1/127 | 0.990 | 1.12 (0.13-2.79) |  |  |  |  | 5/68 | 0.580 | 0.59 (0.09-3.77) |  |  |  |
| Operation time (>600/≤600 minutes) | 12/116 | 0.322 | 1.83 (0.55-6.04) |  |  |  |  | 28/45 | 0.245 | 1.77 (0.68-4.60) |  |  |  |
| Intraoperative blood loss (>1000/≤1000 mL) | 9/119 | 0.068 | 3.80 (0.90-16.00) |  | 0.062 | 4.43 (0.93-21.15) |  | 6/67 | 0.144 | 5.15 (0.57-46.48) |  |  |  |
| Combined MVR (+/-) | 2/126 | 0.691 | 1.74 (0.11-28.47) |  |  |  |  | 9/64 | 0.355 | 2.00 (0.46-8.70) |  |  |  |
| Diameter of the MPD (<3.0/≥3.0 mm) | 59/69 | 0.568 | 0.81 (0.39-1.68) |  | 0.727 | 1.16 (0.51-2.61) |  | - | - |  |  |  |  |
| Pancreatic texture (soft/others) | 111/17 | 0.896 | 1.07 (0.37-3.12) |  |  |  |  | - | - |  |  |  |  |
| ICG-Krem (<0.06/≥0.06) | - | - |  |  |  |  |  | 10/63 | 0.074 | 4.44 (0.87-22.78) |  | 0.365 | 2.29 (0.38-13.81) |
| Preoperative PVE (+/-) | - | - |  |  |  |  |  | 15/58 | 0.912 | 1.07 (0.34-3.33) |  |  |  |
| Type of hepatectomy (right-sided/left-sided) | - | - |  |  |  |  |  | 35/38 | 0.079 | 2.33 (0.91-5.96) |  | 0.097 | 2.57 (0.84-7.83) |
| UICC 7^th^ pT (3-4/1-2) | 51/77 | 0.111 | 1.81 (0.87-3.77) |  |  |  |  | 26/47 | 0.794 | 0.88 (0.34-2.30) |  |  |  |
| Lymph node metastasis (+/-) | 47/81 | 0.156 | 1.71 (0.81-3.58) |  |  |  |  | 24/49 | 0.801 | 1.13 (0.43-3.02) |  |  |  |
| *P* <0.050*  *CCI* comprehensive complication index, *PD* pancreaticoduodenectomy, *Hx* major hepatectomy with extrahepatic bile duct resection, *OR* odds ratio, *CI* confidence interval, *ASA-PS* American Society of Anesthesiologists physical status, *GLIM* global leadership initiative on malnutrition, *MVR* major vascular resection and reconstruction, *MPD* main pancreatic duct, *ICG-Krem,* indocyanine green clearance of remnant liver, *PVE* portal vein embolization, *UICC* Union for International Cancer Control. | | | | | | | | | | | | | |
